# Supplementary material for: The Association of Breakfast Frequency and Cardiovascular Disease (CVD) Risk Factors among Adolescents in Malaysia
Source: Nutrients. 2019 Apr 28;11(5):973. doi: 10.3390/nu11050973 (PMC6567224; doi:10.3390/nu11050973)
Supplement: Supplementary file 1 [file nutrients-11-00973-s001.pdf]

Supplementary table: Food groups and their contents (% reported is the proportion of specific food items out of all items reported consumed for breakfast in that group).

**Food Group name**	**Contents**
Cereal based mixed meals	rice in coconut milk (34%), fried rice (30%), fried rice noodles (12%)
Chocolate and confectionery	granulated sugar (99%)
Hot and powdered drinks	milo (40%), malted milk powder (38%), coffee powder (5%)
High fat milk and cream	sweetened condensed milk (91%), full cream milk (6%)
Bread	white bread (99%)
Other bread products	Indian flatbread (49%), cream crackers (21%)
Rice, pasta and other grains	cooked rice (84%)
Egg and egg dishes	fried eggs (64%), whole egg (32%)
Spreads	egg jam (55%), strawberry jam (18%), peanut butter (11%)
Biscuits and cakes	chocolate cream bread (30%), corn cream bread (11%), vanilla cream bread (8%)
Malaysian kuih (high ED>11.2)	currypuff (50%), doughnuts (24%)
Sauces (high ED > 10 kJ/g)	gravy (75%), chilli paste (11%)
Fish high fat (>4.6g)	fried mackerel (27%), dried fried anchovies (17%)
Meat and poultry	fried chicken (65%), chicken breast (25%)
Sauces (low ED < 10 kJ/g)	chilli sauce (62%), sweet soy sauce (20%)
Oil and fats	butter (56%), margarine (44%)
Sugar-sweetened beverages	squash (27%), cereal drinks (14%), soy drinks (13%)
Meat mixed dishes	chicken fried in chilli (40%), chicken curry (18%), chicken rendang (14%)
Fried vegetables	fried spinach (22%), fried long beans (20%), fried cabbage (20%), fried beansprouts (13%)
Breakfast cereals	koko crunch cereal (60%), rice porridge (9%)
Malaysian kuih (low ED<11.2)	deep fried banana fritters or doughballs (33%), glutinous rice (14%)
Fresh fruit	apples (42%), watermelon (12%)
Soups	noodle soup (41%), chicken soup (7%)
Vegetables (Raw or boiled)	sauté spinach (24%), cabbage (19%), Chinese mustard leaves (18%)
Fish mixed dishes	mackerel cooked in sauce (32%), prawns cooked in chilli (14%), tuna in mayonnaise (13%)
Processed meat	hot dog (45%), sausages (27%), chicken burger (14%)
Low fat milk	low-fat milk (76%)
Coated or breaded meat and fish	chicken nuggets (90%)
Fish low fat (<4.6g)	prawns (29%), fish balls (18%), dried salted fish (14%)
Fruit juice	orange juice (49%), watermelon juice (26%)
Vegetable mixed dishes	cabbage in coconut milk(64%), fiddlehead fern in coconut milk (25%)
Fried/roast potatoes and chips	chips (84%)
Other fruit	raisins (100%)
Cheese	cheddar (100%)
Crisps and savoury snacks	fried fish crackers (57%)
Nuts and seeds	groundnuts (pagoda) (100%)

Meat substitutes	soybean curd (50%), egg soybean curd in oyster sauce (33%)
Legumes	baked beans (50%), red gram (25%)
Puddings	cheese cake (25%), banana pancake (25%), steamed pumpkin parcels (25%)
Cooked potatoes	potatoes (67%)
Ice-creams	chocolate ice cream (100%)
Pizza	pizza (100%)
Water	coconut water (100%)
Yoghurts	yoghurt drink (100%)
